# Targeted Delivery of Exosomes Armed with Anti-Cancer Therapeutics

**DOI:** 10.3390/membranes12010085

**Published:** 2022-01-13

**Authors:** Hojun Choi, Hwayoung Yim, Cheolhyoung Park, So-Hee Ahn, Yura Ahn, Areum Lee, Heekyoung Yang, Chulhee Choi

**Affiliations:** 1 ILIAS Biologics Inc., Daejeon 34014, Korea; hchoi@iliasbio.com (H.C.); hyim@iliasbio.com (H.Y.); chpark@iliasbio.com (C.P.); shahn@iliasbio.com (S.-H.A.); yahn@iliasbio.com (Y.A.); alee@iliasbio.com (A.L.); 2 In Vivo Pharmacology, 1ST Biotherapeutics Inc., Seongnam-si 13493, Korea; heekyoung.yang@1stbio.com; 3 Department of Bio and Brain Engineering, KAIST, Daejeon 34141, Korea

**Keywords:** exosome, cargo loading, targeted delivery, cancer therapeutics, scalable manufacturing

## Abstract

Among extracellular vesicles, exosomes have gained great attention for their role as therapeutic vehicles for delivering various active pharmaceutical ingredients (APIs). Exosomes “armed” with anti-cancer therapeutics possess great potential for an efficient intracellular delivery of anti-cancer APIs and enhanced targetability to tumor cells. Various technologies are being developed to efficiently incorporate anti-cancer APIs such as genetic materials (miRNA, siRNA, mRNA), chemotherapeutics, and proteins into exosomes and to induce targeted delivery to tumor burden by exosomal surface modification. Exosomes can incorporate the desired therapeutic molecules via direct exogenous methods (e.g., electroporation and sonication) or indirect methods by modifying cells to produce “armed” exosomes. The targeted delivery of “armed” exosomes to tumor burden could be accomplished either by “passive” targeting using the natural tropism of exosomes or by “active” targeting via the surface engineering of exosomal membranes. Although anti-cancer exosome therapeutics demonstrated promising results in preclinical studies, success in clinical trials requires thorough validation in terms of chemistry, manufacturing, and control techniques. While exosomes possess multiple advantages over synthetic nanoparticles, challenges remain in increasing the loading efficiency of anti-cancer agents into exosomes, as well as establishing quantitative and qualitative analytical methods for monitoring the delivery of in vivo administered exosomes and exosome-incorporated anti-cancer agents to the tumor parenchyma.

## 1. Introduction

In the past few decades, the field of nanomedicine has experienced unparalleled rapid advances in both disease diagnosis and therapeutic applications [1]. Among novel nanoparticles, extracellular vesicles (EVs) have gained immense attention for their role as therapeutic vehicles for delivering various active pharmaceutical ingredients (APIs) for treating a variety of cancers [2,3]. EVs are natural lipid nanoparticles secreted by almost all types of cells and can be categorized as exosomes, microvesicles, and apoptotic bodies, depending on their biological properties and biogenesis pathway [4,5,6]. Exosomes are composed of a single phospholipid bilayer with a size typically ranging from 30 to 150 nm. They are generally termed for membrane vesicles formed by the inward invagination from endosomes [7]. Exosomes exist in almost all body fluids [8,9,10,11,12,13,14] and actively participate in the intracellular transport of diverse bioactive molecules, such as nucleic acids (DNA and RNA), proteins, and lipids. Microvesicles (or ectosomes) are generated by the direct outward budding of vesicles from the plasma membrane, with sizes ranging from 0.1 to 1 μm [15]. Apoptotic bodies are larger EVs and contain parts of dying cells, such as intact organelles, micronuclei, and chromatin remnants [16]. Different subtypes of EVs have been identified based on their size and density, which allow separation by methods such as tangential flow filtration, size exclusion chromatography, and differential centrifugation [17]. In addition, EVs can be purified via immunoaffinity-based methods by capturing EV-specific markers (e.g., CD81, CD63, CD9 for exosomes) with their corresponding antibodies [17], which could be further extended to isolate disease-specific EVs such as cancer cell-derived exosomes [18]. Nevertheless, careful interpretation is warranted when analyzing different groups of EVs since most EV purification methods cannot distinguish EVs based on their biogenesis pathways, but rather isolate subtypes of EVs based on physical properties [6]. The EV research society recommends clearly stating the definition of the subtypes of EVs in each study [6]. Among the various EVs, microvesicles and exosomes are considered delivery vehicles for diverse biological molecules for intercellular communication.

Numerous technologies to efficiently incorporate anti-cancer drugs and APIs into exosomes are actively being developed [19,20,21]. Engineering strategies for inducing the targeted delivery of exosomes to the tumor parenchyma are being established and validated through various preclinical studies [14]. While exosomes are also widely being investigated as biomarkers for cancer diagnosis [22,23], we will focus on the therapeutic applications of “armed” exosomes for treating various cancers. Here, we highlight the therapeutic potential of exosomes “armed” with various anti-cancer agents and summarize the engineering strategies for the efficient targeted delivery of these “armed” exosomes to tumor cells and the surrounding microenvironments. We also review the critical points that should be considered for the scalable manufacturing of clinical-grade therapeutic exosomes.

## 2. “Armed” Exosomes as Cancer Therapeutics

**Figure 1:**
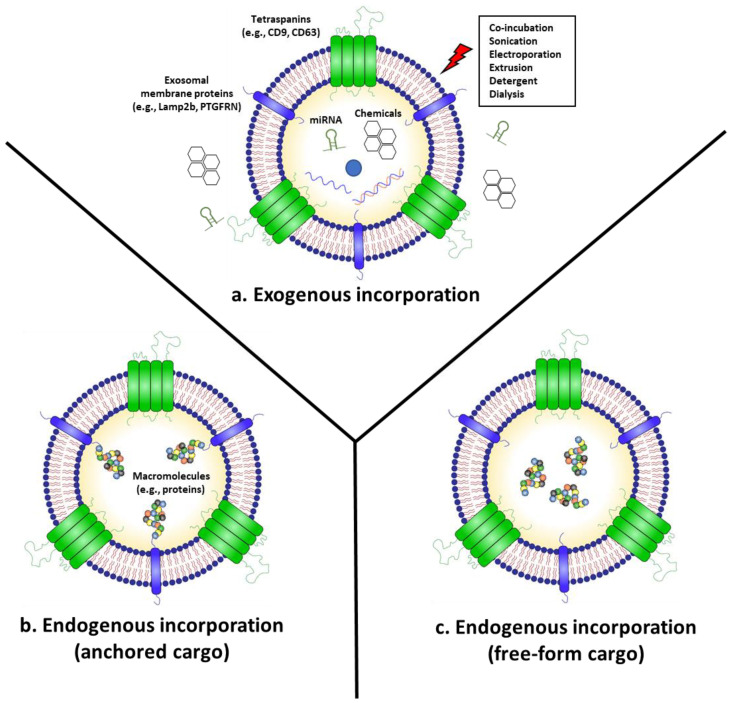
Engineering methods for incorporating therapeutic agents into exosomes. (**a**) Exogenous cargo incorporation can be achieved using methods such as co-incubation, electroporation, sonication, or extrusion to introduce APIs into the exosomes. (**b**,**c**) Endogenous cargo incorporation methods modify the exosome-producing cells to incorporate therapeutic agents into the exosomes through the natural exosome biogenesis pathways. These methods could be divided into two approaches based on whether the cargo is anchored onto the exosomal membrane proteins (e.g., tetraspanins, Lamp2b, PTGFRN) (**b**) or resides as a non-anchored free form inside the lumen of the exosome (**c**).

Naturally produced exosomes, known as naïve exosomes, inherit the physiological characteristics of the cells from which they originate, and show comparable therapeutic potency with a better safety profile, when compared to the original cell therapy [24,25,26]. In addition, many efforts have been made to modify exosomes or exosome-producing cells to incorporate anti-cancer agents into exosomes for advanced cancer therapeutics (Figure 1) [2,3]. Exosomes can incorporate desired therapeutic agents via direct exogenous methods (e.g., electroporation and sonication) or indirect endogenous methods by modifying cells to produce “armed” exosomes. In this section, we review exosomes “armed” with anti-cancer agents and their therapeutic efficacy in eradicating cancer.

### 2.1. Anti-Cancer Exosomes Loaded with RNAs (miRNA, siRNA, and mRNA)

One of the attractive therapeutic approaches for intractable cancers is gene therapy using nucleic acids, such as DNA and RNA. However, there is a need for enhancing their stability for clinical use due to the disruption of the structure and function in the biological fluids. RNA is extremely unstable in plasma and demonstrates low intracellular delivery in vivo, requiring efficient delivery vehicles for therapeutic applications. MicroRNAs (miRNAs) and short-interfering RNAs (siRNAs) are short, non-coding, double-stranded RNAs that suppress gene expression by regulating the translation process of messenger RNAs (mRNAs). Exosomes do not contain large quantities of RNAs, but a wide variety of these nucleic acids—miRNA, ribosomal RNA (rRNA), and mRNA—have been previously detected in isolated exosomes [7,27]. Unmodified naïve exosomes are efficient intracellular delivery vehicles for a wide population of endogenous miRNAs [28], demonstrating their potential as therapeutic carriers of small RNAs [27,29]. Despite the therapeutic potential of the exosome-mediated delivery of mRNA, it is challenging to incorporate long RNAs into exosomes because of their large size. Thus, we will mainly elucidate the role of small RNA-loaded exosomes in anti-cancer therapeutics and will introduce recent advances in loading long RNAs and plasmids into exosomes.

One of the technical approaches for loading small RNAs into exosomes is the transfection of exosome-producing cells with small RNAs, which are then endogenously incorporated into exosomes through their natural biogenesis pathway. Katakowski et al. transfected marrow stromal cells with the miR-146b expression plasmid to obtain miR-146b-loaded exosomes, and the intratumoral injection of these exosomes suppressed glioma growth in a rat xenograft model [30]. Similarly, Ohno et al. transfected HEK293T cells with let-7a miRNA to generate let-7a-loaded exosomes expressing the EGFR-targeting peptide GE11 [31]. These authors also demonstrated the delivery of let-7a to EGFR-expressing breast cancer by an intravenous injection of engineered exosomes in a mouse xenograft model [31]. In addition, O’Brien et al. encapsulated miRNA-379 into mesenchymal stem cell (MSC)-derived EVs by transfecting MSCs with miRNA-379 and observed tumor growth inhibition in an orthotopic mouse model of breast cancer [32]. Nevertheless, the exact exosomal loading mechanism of overexpressed miRNAs and siRNAs has not been fully elucidated in most studies; therefore, it requires further investigation. Interestingly, Hung et al. developed a platform technology called Targeted and Modular EV Loading (TAMEL) to load RNAs into EVs by introducing in exosome-producing cells a fusion protein of a MS2 bacteriophage coat protein that is an RNA-binding protein, and EV-associated proteins such as lysosome-associated membrane glycoprotein 2b (Lamp2b) and CD63 [33]. By expressing MS2 loop-containing cargo RNAs to the engineered cells, exosomes contained up to six-fold higher amounts of cargo RNAs and efficiently delivered loaded RNAs to the cells [33]. While there are no cancer-related applications yet, such new platform technologies could be further applied to produce exosomes loaded with small RNAs for cancer therapeutics. Remarkably, Yang et al. developed a cellular nanoporation method to load mRNAs into exosomes by stimulating mRNA plasmid-transfected cells with transient electric pulses [34]. Using this method, glioma-targeting peptide-labeled exosomes loaded with mRNA coding tumor suppressor phosphatase and tensin homolog (PTEN) inhibited tumor growth and increased survival in an orthotopic mouse model of PTEN-deficient glioma [34].

Small RNAs can also be incorporated into exosomes using exogenous methods, such as electroporation and sonication (Figure 1a) [35,36]. For instance, Kamerkar et al. used electroporation to load siRNAs targeting oncogenic KRAS^G12D^ into exosomes (iExosome) [37]. Upon systemic injection, iExosomes suppressed tumor growth and increased overall survival in multiple mouse models of pancreatic cancer [37]. While there is currently no FDA approved exosomal therapeutics yet, the therapeutic efficacy of iExosomes for pancreatic cancer with KRAS^G12D^ is being evaluated by a phase I clinical trial (NCT 03608631). Similarly, Kase et al. loaded siRNAs targeting lymphocyte cytoplasmic protein 1 (LCP1) into exosomes through electroporation and showed that these exosomes could inhibit the growth of oral squamous cell carcinoma in a mouse xenograft model [38]. Exosomes can also be utilized for the delivery of antisense oligonucleotides (ASOs), which are single-stranded synthetic oligodeoxynucleotides of diverse chemistries [39]. Xu et al. conjugated ASOs with cholesterol for spontaneous insertion into the membrane of exosomes through hydrophobic interactions [40]. HepG2-derived exosomes loaded with G3139, which is an ASO targeting the anti-apoptotic protein Bcl-2, were efficiently delivered to tumor cells and observed to downregulate Bcl-2 in vitro [40]. Large plasmids can also be incorporated into exosomes via electroporation. Kim et al. loaded poly (ADP-ribose) polymerase-1 (PARP-1) targeting CRISPR/Cas9-encoded plasmid vectors into SKOV3 ovarian cancer cell line-derived exosomes by electroporation, reporting an approximately 2% loading efficiency [41]. The loaded SKOV3-derived exosomes demonstrated better targeting to SKOV3 compared to epithelial cell-derived exosomes in a xenograft mouse model and suppressed the expression of PARP-1 and induced apoptosis in ovarian cancer [41].

Despite the promising anti-tumor effects of RNA-loaded exosomes shown in preclinical studies, the therapeutically effective amount of RNAs per exosome needs to be established through further studies. To achieve that goal, the loading amount of RNAs per exosome should be accurately measured to determine the therapeutically effective dose of RNA-loaded exosomes. Determining the loading amount of RNA requires thorough consideration concerning the potential bias caused by different RNA profiling methods, the perturbations made by different exosome purification methods, and the sample-to-sample heterogeneity caused by technical variability [27].

### 2.2. Chemotherapeutics-Armed Exosomes

As exosomes are lowly immunogenic, highly bioavailable, naturally derived lipid nanoparticles, the exosomal incorporation of chemical drugs can enhance the solubility and bioavailability of chemotherapeutics [42]. In addition, exosomes can be engineered to endow a targeted delivery to specific organs and tissues by surface modification [14], which could enhance the delivery of encapsulated drugs to the tumor parenchyma. Similar to small RNAs, anti-cancer chemical drugs can also be incorporated into exosomes using exogenous methods such as co-incubation, electroporation, sonication, and extrusion (Figure 1a) [42]. For instance, Kim et al. compared the loading efficacy of paclitaxel (PTX), a commonly used chemotherapeutic drug with low water solubility, using the following three different methods: co-incubation at room temperature, electroporation, and sonication [43]. The sonication method demonstrated the highest loading efficiency of PTX into the exosomes, and the generated PTX-loaded exosomes inhibited the growth of Lewis lung carcinoma pulmonary metastasis after systemic injection in a mouse xenograft model [43]. PTX-loaded exosomes improved the anti-cancer effects of PTX by increasing its solubility and overcoming Pgp-mediated drug efflux [43]. In addition, Tian et al. loaded doxorubicin (DOX), another commonly used anti-cancer drug with low bioavailability and severe side-effects (e.g., cardiotoxicity), into mouse immature dendritic cell (imDC)-derived exosomes using electroporation [44]. DOX-loaded imDC-derived exosomes expressing αv integrin-specific iRGD peptide suppressed tumor growth of MDA-MB-231 breast cancer cells without severe toxicity in a mouse xenograft model after systemic injection [44]. Gomari et al. also utilized electroporation to encapsulate DOX into MSC-derived exosomes expressing HER2-targeted DARPin [45]. The exosomes demonstrated efficient targeting to HER2-positive breast cancer cells and reduced the tumor growth rate in a mouse xenograft model [45]. Exosomes can also effectively deliver DOX and PTX across the blood–brain barrier (BBB) and into the brain, improving therapeutic efficacy in a xenotransplanted zebrafish model of brain cancer [46]. In addition, Fuhrmann et al. loaded porphyrins with different degrees of hydrophobicity into exosomes via various methods such as electroporation, saponin-assisted drug loading, extrusion, and hypotonic dialysis [47]. Porphyrin-loaded exosomes demonstrated the enhanced cancer cell delivery and photodynamic effects of porphyrin in vitro [47]. Interestingly, loading porphyrin using saponin, a natural detergent, resulted in up to an 11-fold higher loading efficiency compared to co-incubation methods [47]. Recently, McAndrews et al. loaded the STING agonist cyclic GMP-AMP (cGAMP), which activates the cytosolic DNA sensing pathway and subsequent type I interferon (IFN) responses in dendritic cells (DCs), into exosomes via co-incubation at room temperature for 16 h [48]. STING agonist-loaded exosomes suppressed the growth of B16F10 melanoma tumor cells in vivo and demonstrated enhanced delivery to DCs compared with the STING agonist alone, which resulted in an increased recruitment of activated CD8+ T-cells and enhanced anti-tumor immune response [48]. Currently, Codiak Biosciences (Cambridge, MA, USA) is leading a phase I/II clinical trial for testing the efficacy of STING agonist-loaded exosomes as cancer therapeutics (NCT04592484).

Encapsulating chemotherapeutic agents into exosomes confers multiple advantages, such as lowering unwanted toxicity and enhancing tissue targeting. Nevertheless, increasing the loading efficiency of chemotherapeutics into exosomes remains a major challenge, as conventional loading methods such as electroporation and sonication demonstrate loading efficiencies as high as 20% [42]. Exogenous methods also have the risk of disrupting the membrane integrity of exosomes, which could affect their biological functionality or result in unexpected immunogenicity [49].

### 2.3. Exosomes “Armed” with Anti-Cancer Proteins

Macromolecules such as proteins can be incorporated into exosomes using exogenous methods such as electroporation. However, the susceptibility to the destruction of physical structures of proteins and exosomes limits their usefulness, along with the low loading efficiency of functional proteins [5,50]. Hence, engineering exosome-producing cells by genetic modification has been proposed for the active incorporation of therapeutic proteins into exosomes. For instance, exosomes displaying signal regulatory protein α (SIRPα) were generated by transfecting exosome-producing cells with plasmids expressing SIRPα conjugated to the N-terminus of platelet-derived growth factor receptor β (PDGFR-β) [51]. SIRPα-exosomes inhibited tumor growth by binding to and suppressing the function of CD47, a “don’t eat me” signal overexpressed on cancer cells, therefore promoting the engulfment of tumor cells by macrophages [51]. Recently, Dooley et al. developed a platform technology to generate exosomes expressing therapeutic proteins via conjugation with exosomal membrane proteins such as prostaglandin F2 receptor inhibitor (PTGFRN) and brain abundant membrane attached signal protein 1 (BASP1) (Figure 1b) [52]. This platform has been applied to display IL-12 on the surface of exosomes by conjugation to PTGFRN [53]. The generated exosomes triggered the prolonged production of IFN-γ and increased tumor antigen-specific CD8+ T cell responses, which suppressed the growth of MC38 murine colon adenocarcinoma compared with recombinant IL-12 upon intratumoral injection [53]. However, the drawback of membrane-associated approaches is that the therapeutic protein remains anchored to the exosomal membrane after delivery, which may not be applicable for delivering soluble cytosolic or nuclear proteins.

**Figure 2:**
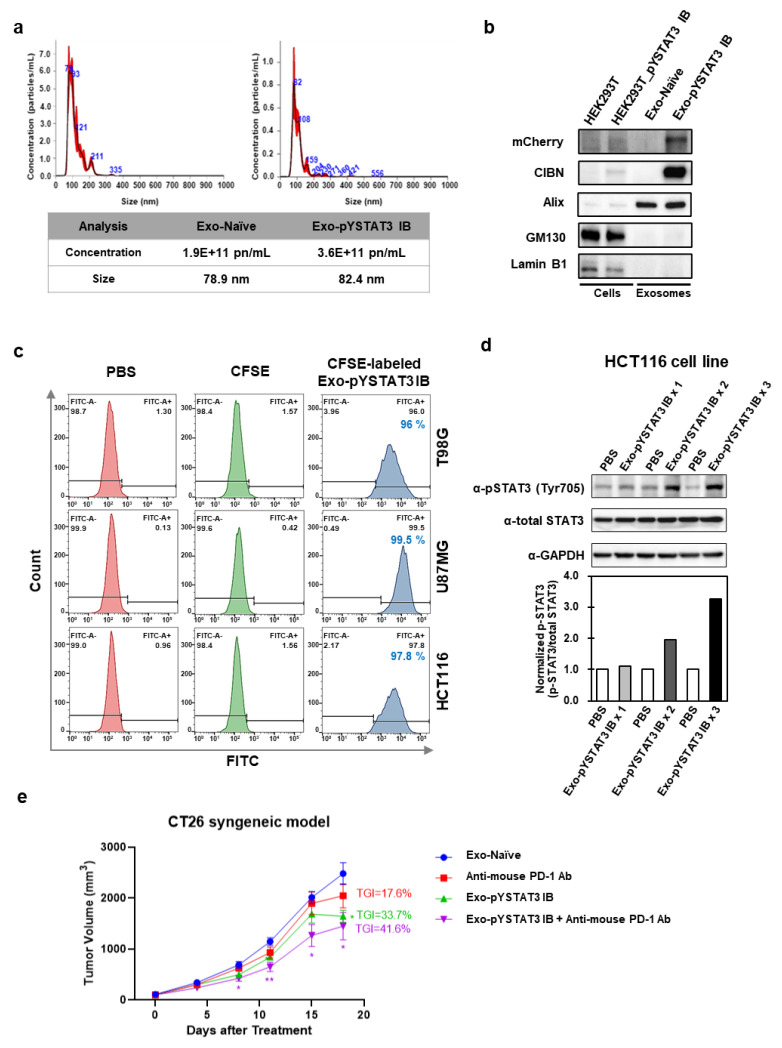
Characteristics of Exo-pYSTAT3 IB and *in vitro* and *in vivo* anti-cancer effects of Exo-pYSTAT3 IB. (**a**) The size and quantification of purified exosomes (Exo-Naïve or Exo-pYSTAT3 IB) from exosome-producing HEK293T cells or HEK293T-mCherry-pYSTAT3 IB cells were analyzed using Nanoparticle tracking analysis (NTA) with a NanoSight NS300. (**b**) The lysates from HEK293T or exosome-producing HEK293T-mCherry-pYSTAT3 IB cells (5 μg) and exosomes (5 × 10^9^ pn) of naïve or pYSTAT3 IB were analyzed using an immunoblot assay. Exo-pYSTAT3 IB (mCherry), EXPLOR^®^ marker (CIBN), exosome positive marker (Alix), exosome negative marker (GM130, Lamin B1). (**c**) A cellular uptake assay of glioblastoma cells (T98G, U87MG) or colorectal cancer cells (HCT116) treated with PBS, CFSE (10 μM), or CFSE-labeled Exo-pYSTAT3 IB (5 × 10^10^ pn) for 24 h were analyzed using FACSCelesta (BD). The exosomes of pYSTAT3 IB were labeled according to the manufacturer’s protocol using CellTrace CFSE Cell Proliferation kit (Thermo). (**d**) HCT116 cells were treated with PBS or 1 × 10^10^ pn/mL of Exo-pYSTAT3 IB (1, 2, or 3 treatment times for 24 h interval) for 24, 48, or 72 h. The lysates (30 μg) of HCT116 cells (PBS or Exo-pYSTAT3 IB) were analyzed using an immunoblot assay. The band intensity of phospho-STAT3 (Tyr705) or total-STAT3 in the immunoblot image was measured and normalized using Image Lab Software (Bio-Rad). (**e**) Female BALB/c mice (6 to 8 weeks of age) were inoculated subcutaneously at the right lower region with CT26 murine colorectal carcinoma cells (3 × 10^5^) in 100 μL of phosphate-buffered saline (PBS). Exo-Naïve (1 × 10^9^), Exo-pYSTAT3 IB (1 × 10^9^), anti-mouse PD-1 antibody (10 mg/kg, RMP1-14, BioXCell, NH, USA), or a combination (Exo-pYSTAT3 IB (1 × 10^9^) and anti-mouse PD-1 antibody (10 mg/kg)) were administered via intraperitoneal injection twice weekly, and treatments were conducted 6 times (D0, D4, D7, D11, D14, and D18). Statistical comparison for tumor growth of different groups was analyzed using a two-way ANOVA followed by Tukey’s post-test for comparing each treatment group (GraphPad Prism 8, San Diego, CA, USA). The tumor volume of the tested groups was expressed as mean ± SEM. *p* values < 0.05 were considered statistically significant. * *p* < 0.05, ** *p* < 0.01.

Alternatively, a platform technology called EXosomes for Protein Loading via Optically Reversible protein–protein interaction (EXPLOR) has been developed, which is able to load non-anchored free-from proteins into exosomes (Figure 1c) [54]. EXPLOR uses blue light-reactive heterodimerizing modules, cryptochrome 2 (CRY2), and the N-terminal of CRY-interacting basic-helix-loop-helix 1 (CIBN) isolated from *Arabidopsis thaliana* [55,56]. By fusing CRY2 with cargo proteins and CIBN with an exosomal membrane protein (CD9), this system allows cargo proteins to be freely loaded in exosomes with high yield, by culturing exosome-producing cells under blue light. Using this EXPLOR platform technology, we have produced engineered exosomes loaded with an mCherry-tagged intracellular antibody (intrabody, IB) targeting a Tyr705-phosphorylated signal transducer and activator of transcription 3 (pYSTAT3). As an oncogene, STAT3 is phosphorylated by the kinases bound to various receptors such as IL-6R and IL-10R. Then, STAT3 is homodimerized and translocated to the nucleus, where it acts as a transcription factor [57,58]. pYSTAT3 IB specifically binds to STAT3 phosphorylated on Tyr705 and blocks its nuclear translocation, thereby inhibiting its function as a transcription factor [59]. Engineered exosomes loaded with pYSTAT3 IB (Exo-pYSTAT3 IB) and naïve exosomes (Exo-Naïve) were analyzed using nanoparticle tracking analysis (NTA), showing that diameters from 80 to 150 nm accounted for over 90% of all exosomes, with a mean size of 82.4 nm and a particle concentration of 3.6 × 10^11^ particle number (pn)/mL for Exo-pYSTAT3 IB (Figure 2a). In contrast to unmodified naïve exosomes, Exo-pYSTAT3 IB was highly loaded with pYSTAT3 IB (Figure 2b). To examine the in vitro cellular uptake of Exo-pYSTAT3 IB in glioblastoma cells (T98G, U87MG) and in colorectal cancer cells (HCT116), we treated cancer cells with CFSE-labeled Exo-pYSTAT3 IB (5 × 10^10^ pn/mL) and analyzed them using flow cytometry (FACS). Almost all the T98G (96%), U87MG (99.5%), and HCT116 (97.8%) cells became CFSE-positive after 24 h of exosome treatment (Figure 2c). Additionally, we observed an accumulation of Tyr705-phosphorylated STAT3 in HCT166 cells after treatment with Exo-pYSTAT3 IB, which is expected to occur via the binding of the delivered pYSTAT3 IB to phosphorylated STAT3 (Figure 2d). The combined administration of anti-mouse PD-1 antibody with Exo-pYSTAT3 IB by intraperitoneal injection resulted in a tumor growth inhibition (TGI) of 41.6% (*p* < 0.05) in a CT26 mouse colon cancer syngeneic model (Figure 2e), suggesting that Exo-pYSTAT3 IB exerts anti-cancer effects by blocking the nuclear translocation of phosphorylated STAT3. These results collectively indicate that “armed” exosomes loaded with anti-cancer protein therapeutics show promising anti-cancer effects.

## 3. Strategies for Targeted Delivery of Exosomes to Cancer Cells

**Figure 3:**
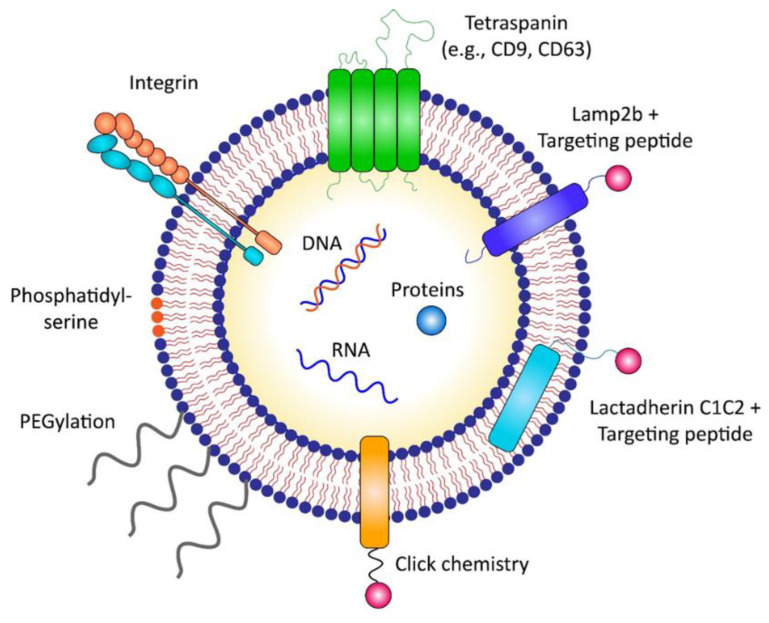
Strategies for targeted delivery of therapeutic exosomes. Targeted delivery of exosomes to cancer cells could be achieved through either “passive” targeting via exosomal membrane proteins such as integrins, or “active” targeting via surface modification. Targeting moieties can be directly labeled to the exosomal surface via various chemical or physical engineering methods, such as click chemistry. In addition, the surface of exosomes can be engineered indirectly by genetically modifying the exosome-producing cells to express targeting peptides fused with exosomal membrane-associated components such as Lamp2b or C1C2 domain of lactadherin.

Anti-cancer therapeutic exosomes could be targeted to cancer cells or tissues in a “passive” or “active” manner (Figure 3). The passive targeting of exosomes occurs through natural tropism, whereas active targeting is achieved via the surface engineering of exosomal membranes using various technical approaches. Generally, exosomes are modified with targeting peptides to demonstrate active targeting to specific organs and cells.

### 3.1. Passive Targeting

Nanoparticles ~100 nm in size are known to be delivered to tumor parenchyma by a mechanism called the “enhanced permeability and retention” (EPR) effect [60,61]. Considering their size, exosomes are also expected to be targeted to tumor tissue after systemic circulation by the ERP effect [62]. Nevertheless, there are debates regarding the extent to which EPR-related phenomena participate in enhancing drug delivery in human cancer tissues, since most of the observations of increased nanoparticle delivery by the EPR effect were based on mouse xenograft models harboring rapidly growing tumors with dense vasculatures. These approaches have limitations in reflecting the tumor microenvironment of most solid tumors in humans [63,64,65,66].

Recent studies have suggested that exosomes can demonstrate natural tumor targetability depending on their cells of origin. Exosomes derived from different parental cells possess different tissue and organ tropisms in vivo [14,67]. Additionally, exosomes derived from tumor cells exhibit efficient targetability to their parental tumor cells, which could be utilized to deliver anti-cancer drugs [68,69,70,71]. Specific exosomal membrane proteins or molecules responsible for selective targetability still need to be elucidated. In an HT1080 xenograft mouse model, HT1080-derived exosomes exhibited as much as twice the delivery efficacy to HT1080 tumors than HeLa-derived exosomes after systemic injection [68]. In a zebrafish xenograft model, DOX and PTX-loaded exosomes originating from brain endothelial cells or brain tumor cells successfully crossed the BBB and demonstrated targeted delivery to brain tumors [46]. Moreover, LNcaP and PC-3 prostate cancer cell line-derived EVs loaded with PTX were effective in delivering PTX to their parental cells [70].

However, Smyth et al. suggested that tumor-derived exosomes harbor tumor targetability only when injected locally into the tumor parenchyma [69]. They observed minimal delivery to the tumor after a systemic administration of breast and prostate tumor-derived exosomes in vivo [69]. Jung et al. also showed that hypoxic cancer cell-derived exosomes demonstrated increased delivery to hypoxic cancer cells only in vitro but not in vivo [71]. More in-depth studies are required to elucidate the mechanism underlying the targetability of tumor cell-derived exosomes to their parental cells. In addition, tumor-derived exosomes may have safety issues in terms of delivering tumorigenic factors to non-tumorigenic cells, which could promote tumor metastasis by inducing the formation of a pre-metastatic niche in potential metastatic sites [72,73,74]. Therefore, careful consideration is needed when using tumor-derived exosomes as delivery vehicles for anti-cancer agents. Instead, understanding the tumor targeting mechanism of tumor-derived exosomes can help design more efficient targeting strategies. For instance, exosomes displaying integrin α6β4 and α6β1 are targeted to laminin-enriched lung microenvironments, especially S100-A4-positive fibroblasts and surfactant protein C-positive epithelial cells [75]. In contrast, exosomes expressing integrin αvβ5 are targeted to fibronectin in the liver microenvironment, which are specifically delivered to F4/80 positive Kupffer cells [75]. In addition, Qiao et al. identified eight different integrins (integrin αv, α3, α5, α6, β1, β4, β5, and β6) in tumor-derived exosomes using proteomic analysis, suggesting that these integrins may participate in conferring tumor tropism to tumor-derived exosomes [68]. In addition, the delivery of glioblastoma (GBM) cell-derived EVs to the recipient GBM cells was shown to involve a triple interaction between the chemokine receptor CCR8 expressed on cells, the glycans exposed on GBM-derived EVs, and the soluble ligand CCL18, which, in turn, promoted the proliferation of GBM cells and resistance to the alkylating agent temozolomide [76].

### 3.2. Active Targeting

Active targeting of anti-cancer therapeutic exosomes to cancer cells or tissues could be achieved by the following two main strategies: one is a non-genetic approach that directly engineers the surface of exosomes via diverse exogenous methods, and the other is a genetic approach that non-directly engineers exosomes by genetically modifying exosome-producing cells (Table 1) [77,78,79].

#### 3.2.1. Direct Surface Engineering of Exosomes

The surface of exosomes can be directly modified via various chemical or physical engineering methods. Targeting moieties can be labeled to the exosomal surface either by covalent attachment methods such as “click chemistry” or through non-covalent methods [14,80]. Click chemistry, also termed copper-catalyzed azide-alkyne cycloaddition, is a highly efficient reaction between an alkyne and an azide residue to form a stable triazole linkage, which can be applied to attach targeting moieties on the surface of exosomes [80,81,82,83,84]. Jia et al. conjugated neuropilin-1 (NRP-1) targeting peptide (RGE peptide) to the exosomal membrane using click chemistry, which promoted glioma targeting and BBB penetration in orthotopic glioma models since NRP-1 was reported to be overexpressed in glioma cells and tumor vascular endothelium [85,86]. PEGylation, which is the chemical conjugation of drugs with branched polyethylene glycol (PEG), is one of the most common modifications used in nanoparticles such as exosomes to generate “armored” nanoparticles for increasing circulation time by evading phagocytosis [87]. “Armored” exosomes modified with aminoethyl anisamide-PEG (AA-PEG) exhibited enhanced delivery to sigma receptor-overexpressing lung cancer by AA-PEG functioning as a targeting ligand for the sigma receptor, a membrane-bound protein with an undefined role [88]. Koojimans et al. generated PEGylated EVs expressing epidermal growth factor receptor (EGFR) nanobodies by mixing EVs with micelles composed of phospholipid (DMPE)-PEG derivatives conjugated with EGFR nanobodies [89]. Armored EVs demonstrated increased binding to EGFR-overexpressing tumor cells in vitro, as well as prolonged circulation time in vivo [89]. However, the drawback of the direct exogenous modification of exosomes is that they mostly require the use of toxic chemicals for exosomal surface engineering, thus raising caution for application in clinics.

#### 3.2.2. Indirect Engineering of Exosomes by Modifying Exosome-Producing Cells

The surface of exosomes can be engineered indirectly by genetically modifying exosome-producing cells. The indirect engineering of exosomes has several advantages over direct surface modification in terms of the expression yield and stability of targeting moieties displayed on exosomes [90,91]. The genetic modification of exosome-producing cells is achieved by transfecting plasmids expressing targeting moieties (e.g., peptides, receptors, and antibodies) fused to exosomal membrane-associated components such as tetraspanins (e.g., CD9, CD63), Lamp2b, and the C1C2 domain of lactadherin [90,92]. For instance, anti-HER2 single-chain variable fragments (scFv) were expressed on the surface of exosomes via conjugation with the C1C2 domain of lactadherin, which is localized to the outer exosomal membrane by interaction with phosphatidylserine [93]. In addition, the fusion proteins of anti-EGFR nanobodies were conjugated with glycosylphosphatidylinositol (GPI)-anchor signal peptides to induce the targetability of exosomes to EGFR-expressing tumor cells [94]. Exosomes are enriched in lipid raft-associated lipids and proteins, including GPI and GPI-anchored proteins, which enable exosomal surface labeling by anchoring to GPIs [95]. Compared to naïve exosomes, HER2- and EGFR-targeting exosomes demonstrated enhanced delivery to HER2- or EGFR-expressing breast cancer cells in vitro, with approximately 2-fold and ~2–3-fold higher accumulation, respectively [93,94]. In addition, αv integrin-targeting iRGD peptide fused with Lamp2b was expressed on the surface of exosomes produced from imDCs, and these exosomes were incorporated with DOX as an anti-cancer agent via electroporation [44]. Compared to unmodified exosomes, iRGD peptide-labeled exosomes demonstrated a three-fold increased cellular uptake by human breast cancer cells in vitro and exhibited enhanced anti-tumor efficacy in vivo with a three-fold decrease in tumor volume [44]. Similarly, the non-small cell lung cancer (NSCLC)-homing peptide, Tlyp-1, was expressed on the exosomal surface by conjugation with Lamp2b for a targeted delivery to human lung cancer cells, showing a two-fold increase in the delivery of Tlyp-1-labeled exosomes to A549 NSCLC tumor cells [96]. Gomari et al. fused HER2 targeting DARPins (designed ankyrin repeat proteins) to Lamp2b to target HER2-positive breast cancer, which resulted in a four-fold increased delivery of engineered exosomes to HER2 positive BT-474 breast cancer cells in vitro [97]. In addition, Apo-A1 was conjugated with CD63, a tetraspanin family used as an exosome marker, for targeted delivery to HepG2 by binding to scavenger receptor class B type 1 expressed on HepG2, showing a two-fold increased uptake of engineered exosomes in vitro [98]. Gong et al. also developed a strategy to target triple-negative breast cancer by expressing a disintegrin and metalloproteinase 15 (A15) on the membrane of exosomes [99]. A15 binds to integrin αvβ3 in an RGD (Arg-Gly-Asp)-dependent manner, which could target αvβ3 overexpressing tumors such as melanoma, glioma, and breast cancer [100,101,102]. Yang et al. used two different peptides, the CDX peptide for U87 glioblastoma targeting and the CREKA peptide for GL261 glioma targeting. Each of these peptides was inserted into the N terminus of CD47 for expression on the surface of exosomes [34]. CDX-labeled exosomes loaded with *PTEN* mRNA by cellular nanoporation demonstrated a two-fold increased accumulation in orthotopically implanted U87 glioma in nude mice and prolonged survival with a median survival of 49 days, compared with the 37 days median survival reported for non-engineered exosomes [34]. In addition, CREKA-labeled exosomes loaded with *PTEN* mRNA showed 1.5-fold higher accumulation in orthotopically implanted U87 glioma in C57BL/6 mice and increased survival with a median survival of 45 days, compared with 31 days for non-engineered exosomes [34].

Interestingly, Kamerkar et al. generated KRAS^G12D^ targeting siRNA-loaded iExosomes expressing high levels of CD47 (CD47^high^ exosomes) by overexpressing CD47 in normal fibroblast-like mesenchymal cells to generate “armored” exosomes that are able to evade phagocytosis by monocytes [37]. CD47^high^ exosomes exhibited a two-fold increased retention in the circulation of mice 3 h after an intraperitoneal injection compared to CD47 knockout exosomes, the retention of which was abrogated by anti-CD47 antibody blocking the interaction of CD47 with SIRPα but not with the non-blocking anti-CD47 antibody [37].

Overall, expressing specific cancer-targeting moieties on the surface of exosomes by conjugation with exosomal membrane-associated domains such as GPI, C1C2 domain, Lamp2b, and tetraspanins, could serve as a promising strategy for the active targeting of anti-cancer therapeutic exosomes to cancer cells and tissues.

**Table 1:** Engineering strategies for targeted delivery of therapeutic exosomes to cancer cells.

Category	Method	Targeting Moiety	Target Cancer	Ref.
Direct engineering of exosomes	Click chemistry	Neuropillin-1-targeting RGE peptide (RGERPPR)	Glioma	[85]
	PEGylation	Aminoethyl anisamide-PEG (AA-PEG)	Sigma receptor-overexpressing lung cancer	[88]
	Mixing with micelles	DMPE-PEG conjugated with anti-EGFR nanobody	EGFR-overexpressing tumor cells in vitro	[89]
Indirect engineering of exosomes	Conjugation with C1C2 domain	Anti-Her2 scFv	HER2-expressing breast cancer	[93]
	GPI anchorage	Anti-EGFR nanobody	EGFR-expressing breast cancer	[94]
	Conjugation with Lamp2b	α_v_ integrin-targeting iRGD peptide	Breast cancer cell line	[44]
		NSCLC-homing peptide Tlyp-1	Lung cancer cell line	[96]
		HER2 targeting DARPins	HER2-expressing breast cancer	[97]
	Conjugation with CD63	Apo-A1	Hepatocellular carcinoma	[98]
	Conjugation with CD47	U87-targeting CDX peptide, GL261-targeting CREKA peptide	U87 glioblastoma cell, GL261 glioma cell	[34]

## 4. Scalable Manufacturing of GMP-Grade Therapeutic Exosomes

As exosomes are cell-derived nanoparticles for the delivery of therapeutics, considerations including the selection of proper exosome-producing cells, the development of efficient cargo loading technology, and the establishment of large-scale production and purification systems should be made for the successful entry of exosomes onto the clinical stage. The choice of exosome-producing cells should be considered very carefully to minimize the incorporation of unwanted cellular bioactive cargoes and maximize the loading of the desired therapeutic payloads on a large scale. To increase the cell culture capacity and exosome productivity, suspension cells are preferred for exosome-producing cells because of their feasibility in applying large-scale culture technologies such as various types of bioreactors [103,104]. Moreover, robust chemistry, manufacturing, and control (CMC) techniques should be established to produce large quantities of good manufacturing practice (GMP)-grade exosomes suitable for clinical trials [105,106]. Culturing methods for exosome-producing cells should be carefully considered and monitored to generate therapeutic exosomes with low immunogenicity, high scalability, and consistency [107,108]. Additionally, an advanced analysis, characterization, purification, and evaluation of exosomes are needed to minimize batch-to-batch variance due to the high heterogeneity of EVs [109]. In addition, technologies to detect and eliminate diverse impurities originating from host cells and non-exosomal EVs should be established and applied during the manufacturing process to produce safe and pure exosome-based therapeutics [110,111]. The currently available exosome separation and purification strategies include ultracentrifugation, size-based separation using membrane filters, precipitation-based separation using polymer and protein organic solvents, ion-exchanged chromatography based on electrostatic interactions, among others [108,112,113,114,115]. However, some limitations should be overcome in terms of isolation efficiency, feasibility, low yield, cost efficiency, and the mass productivity of clinical-grade exosomes [116].

## 5. Conclusions

Among anti-cancer nanomedicines, lipid-based nanoparticles such as liposomes and polymeric micelles constitute the majority of clinically approved therapeutics [117]. Liposomes encapsulating chemical therapeutics such as doxorubicin (DOX) were the first to achieve clinical approval as cancer nanomedicines (Doxil, approved by the FDA in 1995) [118]. In addition, inorganic nanoparticles (e.g., gold particles [119] and iron oxide nanoparticles [120]) are also being investigated for their anti-cancer efficacy. Of those, iron oxide nanoparticle NanoTherm [120] and hafnium oxide nanoparticle Hensify [121] have received clinical approval. Exosomes have comparable advantages over synthetic nanoparticles for low immunogenicity, enhanced intracellular delivery efficacy, and efficient engineering for inducing targetability to specific organs and tissues. Anti-cancer agent-armed exosomes possess great potential not only for the efficient intracellular transport of anti-tumor APIs but also for the enhanced targetability to tumor burden. Exosomes can incorporate desired therapeutic molecules via exogenous (e.g., electroporation, sonication) or endogenous (by modifying exosome-producing cells) methods. Recent advances in cargo-loading platform technologies for generating therapeutic exosomes have allowed the efficient incorporation of macromolecular anti-cancer agents into exosomes [50]. While preclinical studies have shown promising results regarding the therapeutic potential of anti-cancer exosomes, advances in clinical applications of exosome therapeutics will need thorough consideration in terms of CMC due to the heterogeneity of exosomes [106]. For the successful development of clinically approved exosome therapeutics for cancer, establishing methods for the accurate measurement of APIs in exosomes would be needed to determine therapeutically effective doses of exosomes. Tracking the fate of administered exosome-loaded APIs in vivo is quite a different problem from tracking the exosome itself, as labeling the exosomal surface with imaging agents might result in tracking a ghost, not the therapeutic cargo. Thus, methods for the quantitative and qualitative monitoring of exosomes as well as exosome-incorporated anti-cancer agents to the tumor parenchyma should be developed.

## Data Availability

The datasets generated and/or analyzed during the current study are available from the corresponding author upon reasonable request.
